# Insecticidal Efficacy of Silicon Dioxide as Grain Protectant and Surface Treatment Against Two Major Stored-Product Beetle Species

**DOI:** 10.3390/biology15191772

**Published:** 2026-10-06

**Authors:** Maria K. Sakka, Eirini Karanastasi, Sofia Andreadaki, Georgia V. Baliota, Marianna Rigopoulou, Paraskevi Agrafioti, Christos G. Athanassiou, Christos I. Rumbos

**Affiliations:** 1 Laboratory of Entomology and Agricultural Zoology, Department of Agriculture, Crop Production and Rural Environment, University of Thessaly, 38446 Volos, Greece; msakka@uth.gr (M.K.S.); mpaliota@uth.gr (G.V.B.); mrigopoulou@uth.gr (M.R.); agrafiot@uth.gr (P.A.); athanassiou@uth.gr (C.G.A.); 2 Laboratory of Plant Protection, Department of Agriculture, University of Patras, Messolonghi University Campus, 30200 Messolonghi, Greece; ekaranastasi@upatras.gr (E.K.);; 3 Department of Agriculture, International Hellenic University, Alexander Campus, 57400 Thessaloniki, Greece

**Keywords:** desiccant dust, food storage, pest management, residual activity, stored-product protection

## Abstract

The insecticidal efficacy of a commercial silicon dioxide (SiO_2_) formulation (ChinCheX^®^) was evaluated as a grain protectant and surface treatment against adults of *Oryzaephilus surinamensis* (L.) and *Tribolium confusum* Jacquelin du Val. In grain-treatment bioassays, wheat and barley kernels were treated with 0, 100, 200, 500 and 1000 ppm of the SiO_2_ formulation and adult mortality was assessed after 3, 7, 14 and 21 d of exposure. Mortality increased with both concentration and exposure interval. Complete mortality of *O. surinamensis* was achieved at all concentrations, including 100 ppm after 21 d, whereas *T. confusum* was less susceptible at the lowest concentration but reached complete mortality at concentrations of 200 ppm or higher. Progeny production of both species was almost completely suppressed in all treated groups. In surface-treatment bioassays on concrete and plastic surfaces, high mortality (>85%) was recorded within 3 d of exposure at all application rates, while complete mortality was generally achieved within 7 d regardless of surface type. Overall, the SiO_2_ formulation tested exhibited strong insecticidal activity both as a grain protectant and as a surface treatment, indicating considerable potential for the management of storage beetles in stored-product protection programs.

## Simple Summary

Stored-product beetles can cause serious losses in stored grains and related commodities; therefore, there is increasing interest in alternative control methods that are effective and suitable for use in storage facilities. In this study, we evaluated a commercial silicon dioxide formulation, ChinCheX^®^, against two important stored-product beetles, the saw-toothed grain beetle, *Oryzaephilus surinamensis*, and the confused flour beetle, *Tribolium confusum*. The product was tested both as a grain protectant on wheat and barley, as well as surface treatment on concrete and plastic surfaces. Mortality increased with concentration and exposure time, while progeny production was almost completely suppressed in treated grains. The formulation was also highly effective when applied to surfaces, causing rapid mortality of both species. These results indicate that ChinCheX^®^ has strong potential as a grain and surface treatment for the management of stored-product beetles.

## 1. Introduction

Insect infestations in stored commodities are a major cause of post-harvest losses, affecting both the quantity and commercial quality of cereals and other durable food products [1,2,3]. The saw-toothed grain beetle, *Oryzaephilus surinamensis* (L.) (Coleoptera: Silvanidae), and the confused flour beetle, *Tribolium confusum* Jacquelin du Val (Coleoptera: Tenebrionidae), are common pests of stored cereals, where they infest grain masses, flour, and other food commodities, resulting in contamination, weight loss, and reduced market value [4,5].

Management of stored-product insects relies heavily on chemical control, including fumigants and residual insecticides [6,7]. However, the increasing occurrence of insecticide resistance, together with regulatory restrictions on several active ingredients and growing concerns regarding food safety and environmental impact, has intensified the search for alternative control methods. Consequently, integrated pest management (IPM) programs increasingly incorporate non-conventional control tools that are effective, environmentally compatible, and suitable for long-term use [8,9,10,11,12,13].

Among these alternatives, inert dusts have attracted considerable attention [14,15,16]. Diatomaceous earth (DE) and amorphous silica-based formulations act primarily through disruption of the insect cuticle, leading to water loss and eventual death by desiccation [17]. Unlike conventional neurotoxic insecticides, these materials act physically rather than chemically, causing insect mortality through desiccation. Numerous studies have demonstrated the efficacy of DE and silica-based products against a wide range of stored-product insect species under laboratory and field conditions [18,19,20].

Recently, amorphous silica formulations have gained renewed interest because of their high insecticidal activity and relatively low application rates. ChinCheX^®^ is a commercial silicon dioxide (SiO_2_) formulation originally developed for the control of bed bugs, such as *Cimex lectularius* L. (Hemiptera: Cimicidae). A recent study showed that the same formulation is also effective when applied to grain, causing high adult mortality and substantially reducing progeny production in the granary weevil, *Sitophilus granarius* (L.) (Coleoptera: Curculionidae), and the lesser grain borer, *Rhyzopertha dominica* (F.) (Coleoptera: Bostrichidae) [21]. These findings suggest that amorphous silica formulations may have considerable potential for broader application against stored-product pests.

Despite these promising results, information on the efficacy of ChinCheX^®^ against secondary stored-product beetles remains limited. In particular, no data are currently available regarding its effectiveness against *O. surinamensis* and *T. confusum* when applied either directly to grain or as surface treatment on storage-associated surfaces. Therefore, the objective of the present study was to evaluate the insecticidal efficacy of ChinCheX^®^ against adults of *O. surinamensis* and *T. confusum* under laboratory conditions at different concentrations ranging from 100 to 1000 ppm. The formulation was evaluated in two application scenarios: on wheat and barley kernels as a grain protectant, and on concrete and plastic surfaces as a surface treatment. In both cases, adult mortality and subsequent progeny production were assessed.

## 2. Materials and Methods

### 2.1. Ιnsects and Insecticide Formulation

Colonies of *O. surinamensis* and *T. confusum* used in this study were maintained at the Department of Agriculture, University of Patras (Messolonghi, Greece). The insects were reared at 25 ± 1 °C and 65% relative humidity under continuous darkness. *Oryzaephilus surinamensis* was maintained on oat flakes, whereas *T. confusum* was reared on wheat flour. Adult beetles younger than one month were used in all bioassays. The commercial silicon dioxide formulation ChinCheX^®^ Bed Bugs Insecticide (Fruit Tree Limited, Chai Wan, China; 99% amorphous silica dust) was tested. All concentrations and application rates refer to the amount of the commercial formulation applied to the grain or surfaces, respectively.

### 2.2. Bioassay I: Grain Treatment

The first bioassay series evaluated the SiO_2_ formulation as a grain treatment against *O. surinamensis* and *T. confusum*. Wheat (var. Mexa) and barley kernels (var. Persephone) were used. Grain moisture was measured with a Multitest moisture meter (Gode SAS, Le Catelet, France) and ranged from 12 to 13%. For each concentration and replicate, 500 g of grain was placed in 1-L glass jars and treated with 100, 200, 500, or 1000 ppm of the formulation (mg/kg grain). An additional set of untreated jars was included as the control (0 ppm). The jars were manually shaken for approximately 2 min to promote uniform distribution of the dust throughout the grain mass.

Three 20-g samples were taken from each jar and transferred to cylindrical plastic vials (Rotilabo^®^ sample tins with snap-on lids; Carl Roth GmbH & Co. KG, Karlsruhe, Germany; 3 cm in diameter and 8 cm in height). A central opening in each lid was covered with fine mesh, while the inner neck of each vial was coated with Fluon (Northern Products, Woonsocket, RI, USA) to prevent insect escape. Twenty adults of one species were introduced into each vial, with separate vials used for the two species. The vials were maintained at 25 °C and 65% relative humidity under continuous darkness. Mortality was assessed after 3, 7, 14, and 21 d, and dead adults were removed after each assessment. Following the final assessment, all adults, both alive and dead, were removed, and the vials were returned to the incubators under the same conditions for an additional 65 d. At the end of this period, the vials were opened and all progeny, whether alive or dead, were counted. The experiment was repeated three times using freshly prepared treated and untreated grain lots for each replicate. Within each replicate, three vials were prepared for every concentration–species combination, resulting in 3 × 3 vials per treatment.

### 2.3. Bioassay II: Surface Treatment

The second bioassay series examined the efficacy of the formulation when applied to surfaces. Four application rates were tested: 2.5, 5.0, 10.0, and 20.0 g/m^2^, corresponding to 15.5, 31.0, 62.0, and 124.0 mg per Petri dish, respectively. The assays were conducted in standard plastic Petri dishes measuring 89 mm in diameter and providing a surface area of 62 cm^2^. The dishes contained either a concrete-covered surface or an untreated plastic surface.

For the concrete treatments, concrete was mixed with tap water at a 4:1 ratio and stirred thoroughly to obtain a homogeneous paste. Approximately 15 mL of the mixture was placed in each dish and allowed to dry for 24 h. The required amount of SiO_2_ was then weighed with a precision balance and evenly distributed over the surface by gently rotating and tilting the dish. Untreated dishes served as controls (0 g/m^2^). The inner walls of all dishes were coated with Fluon to prevent insect escape. Twenty adults of each species were introduced into separate dishes, and approximately five wheat kernels were provided as food. The dishes were maintained at 25 °C and 65% relative humidity under continuous darkness. Mortality was recorded after 3, 7, 14, and 21 d and was confirmed by gently probing the insects with a fine brush; individuals that did not move were considered dead. Each treatment included three replicate dishes, and the entire experiment was repeated three times using newly prepared treated and untreated dishes, resulting in 3 × 3 dishes per treatment.

### 2.4. Statistical Analysis

Mortality data were analyzed separately for each insect species using repeated-measures MANOVA. Exposure time (days) was treated as the repeated-measures factor, mortality percentage as the response variable, and commodity and insecticide concentration as the main effects. Before analysis, the data were checked for normality and homogeneity of variance, and mortality percentages were analyzed without transformation. Differences among concentrations were evaluated within each species, commodity, and exposure interval using Tukey–Kramer HSD post hoc tests at α < 0.05. The same analytical approach was applied to the surface-treatment data. Progeny production was analyzed using a Poisson generalized linear model including insect species, concentration, commodity, and their interactions. When significant effects were detected, pairwise comparisons of model-estimated means were conducted using Tukey–Kramer HSD tests at α = 0.05. All analyses were performed with JMP Student Edition 18.2.1 (JMP Statistical Discovery LLC, Cary, NC, USA) [22].

## 3. Results

### 3.1. Commodity Treatments

Repeated-measures MANOVA indicated that mortality in both *O. surinamensis* and *T. confusum* varied significantly according to commodity type, insecticide concentration, and exposure time (Table 1). Only two non-significant interaction effects were observed. The commodity × concentration interaction was not significant for *O. surinamensis*, whereas the time × commodity interaction was not significant for *T. confusum*, indicating that mortality on the different commodities did not change significantly over the exposure period in this species.

**Figure 1:**
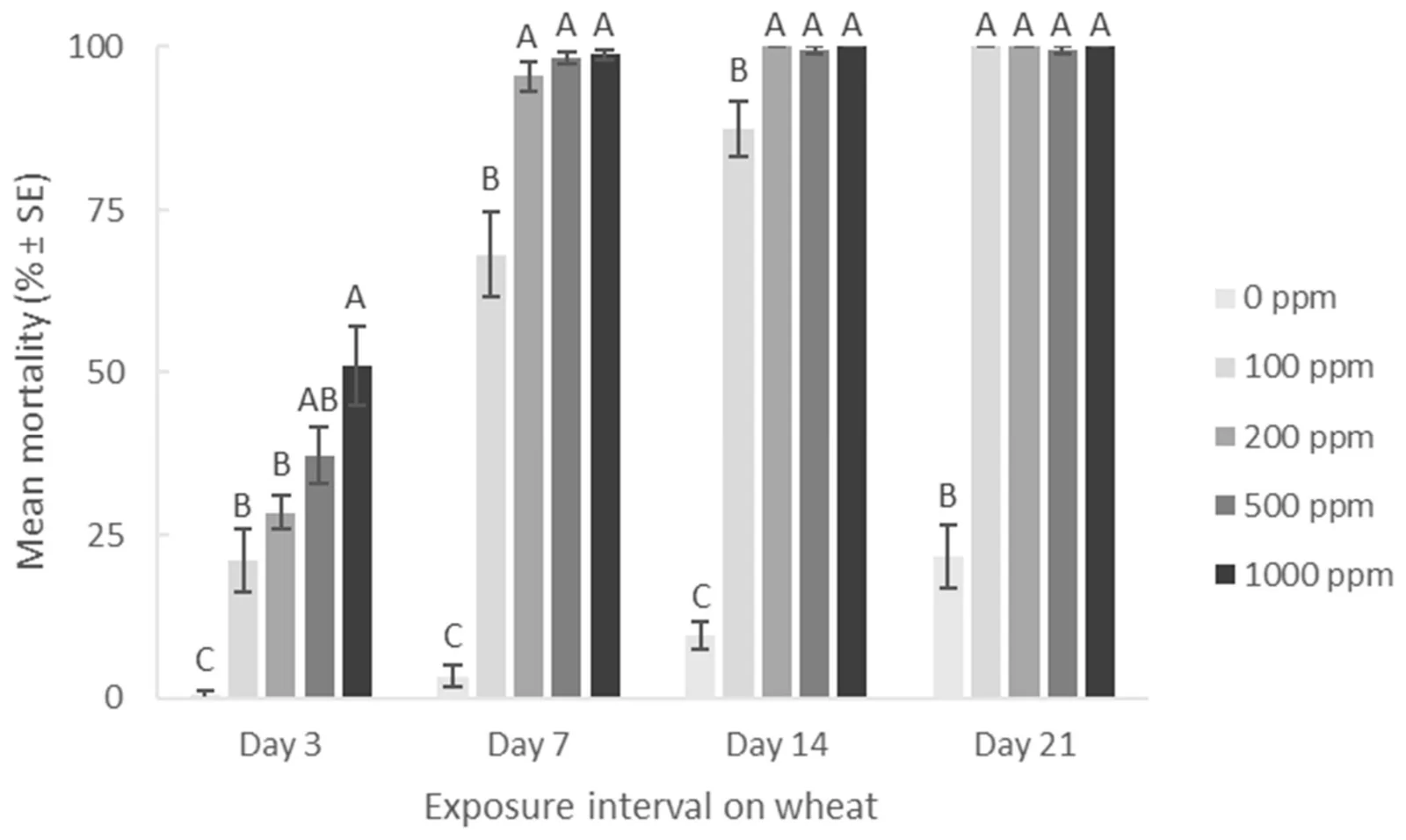
Mean mortality (% ± SE) of *O. surinamensis* after 3, 7, 14 and 21 d of exposure to wheat treated with 0, 100, 200, 500 and 1000 ppm of ChinCheX^®^. Different letters among concentrations within each exposure interval indicate significant differences according to Tukey’s HSD test at α = 0.05.

**Figure 2:**
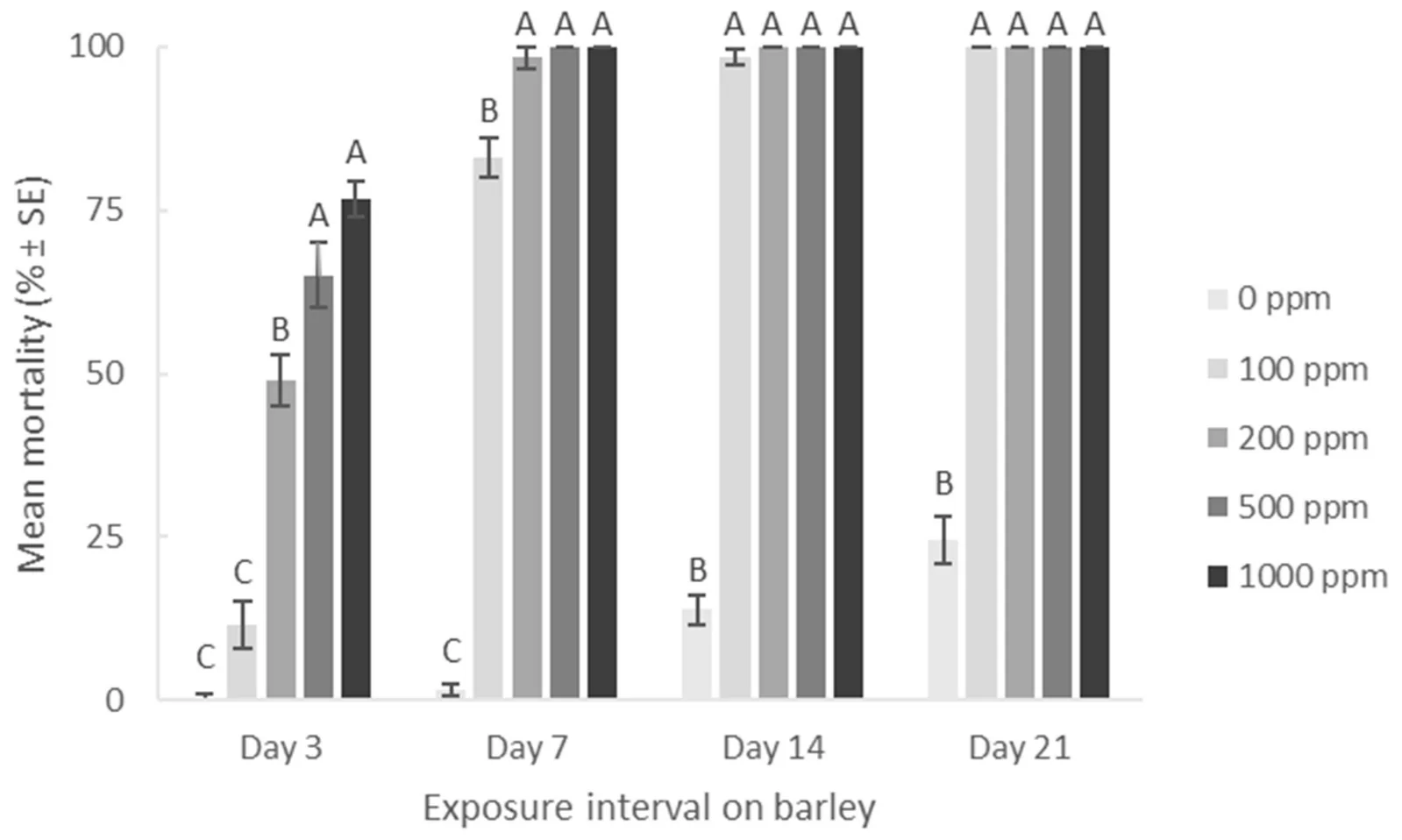
Mean mortality (% ± SE) of *O. surinamensis* after 3, 7, 14 and 21 d of exposure to barley treated with 0, 100, 200, 500 and 1000 ppm of ChinCheX^®^. Different letters among concentrations within each exposure interval indicate significant differences according to Tukey’s HSD test at α = 0.05.

Mortality of *O. surinamensis* on wheat and barley at the different concentrations and exposure intervals is presented in Figure 1 and Figure 2, respectively. Mortality of *O. surinamensis* on wheat increased with both insecticidal concentration and exposure interval (Figure 1). Mortality in the control treatment (0 ppm) remained low throughout the experiment. Even the lowest concentration tested (100 ppm) resulted in 100% mortality after 21 d of exposure, whereas higher concentrations achieved complete mortality within shorter exposure intervals. Specifically, 100% mortality was reached after 14 d at 200 ppm and after only 7 d at 500 and 1000 ppm. Post hoc comparisons showed significant differences among concentration treatments up to 14 d of exposure. However, mortality at 200, 500, and 1000 ppm reached similarly high levels after 7 d, indicating no significant differences among the highest concentrations thereafter (Figure 1). A similar pattern was observed for barley (Figure 2), with the 500 and 1000 ppm treatments causing significantly higher mortality of *O. surinamensis* than the 100 and 200 ppm treatments during the first 3 d of exposure. By 14 d, however, all concentration treatments produced complete or nearly complete mortality.

**Figure 3:**
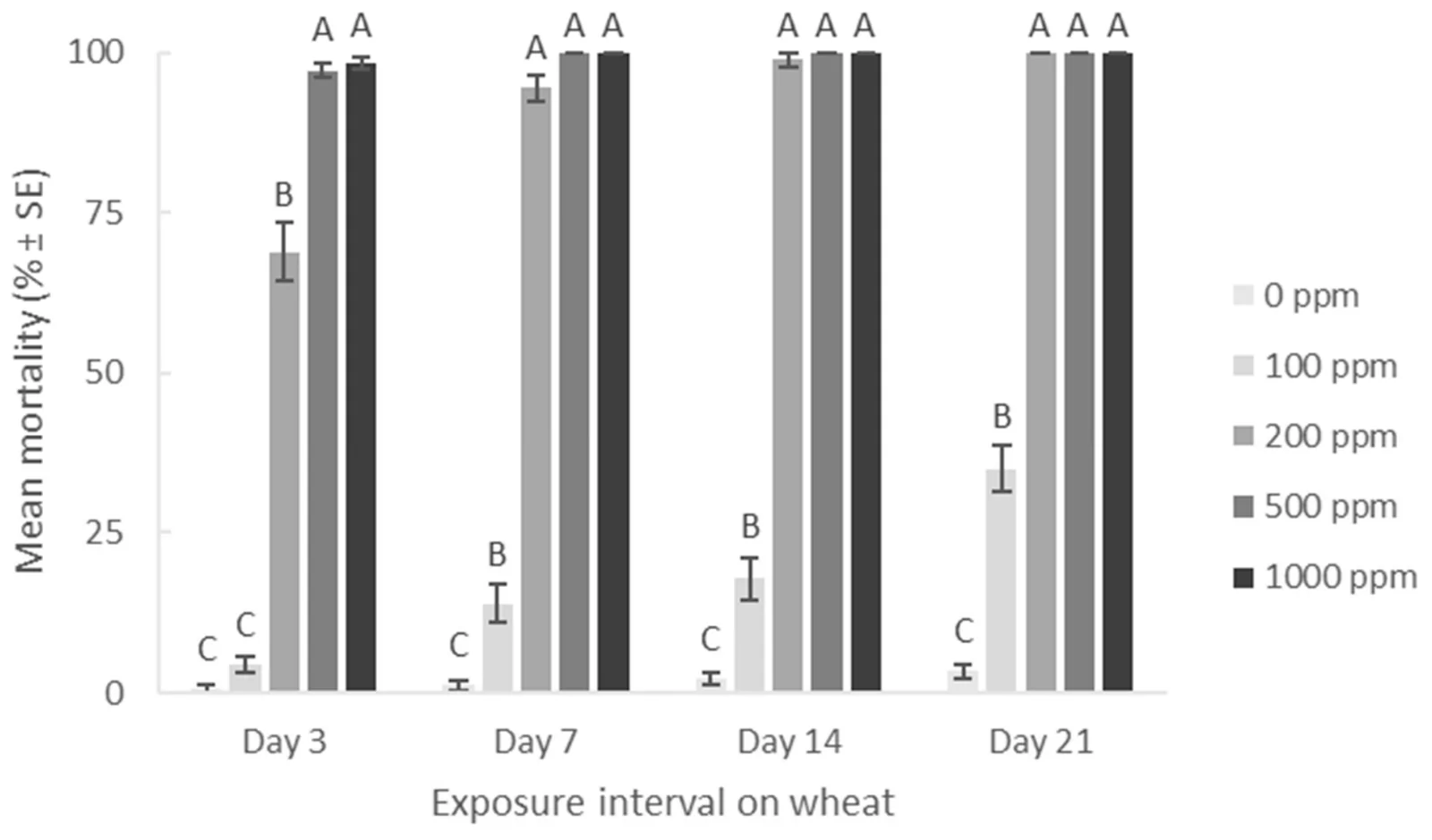
Mean mortality (% ± SE) of *T. confusum* after 3, 7, 14 and 21 d of exposure to wheat treated with 0, 100, 200, 500 and 1000 ppm of ChinCheX^®^. Different letters among concentrations within each exposure interval indicate significant differences according to Tukey’s HSD test at α = 0.05.

**Figure 4:**
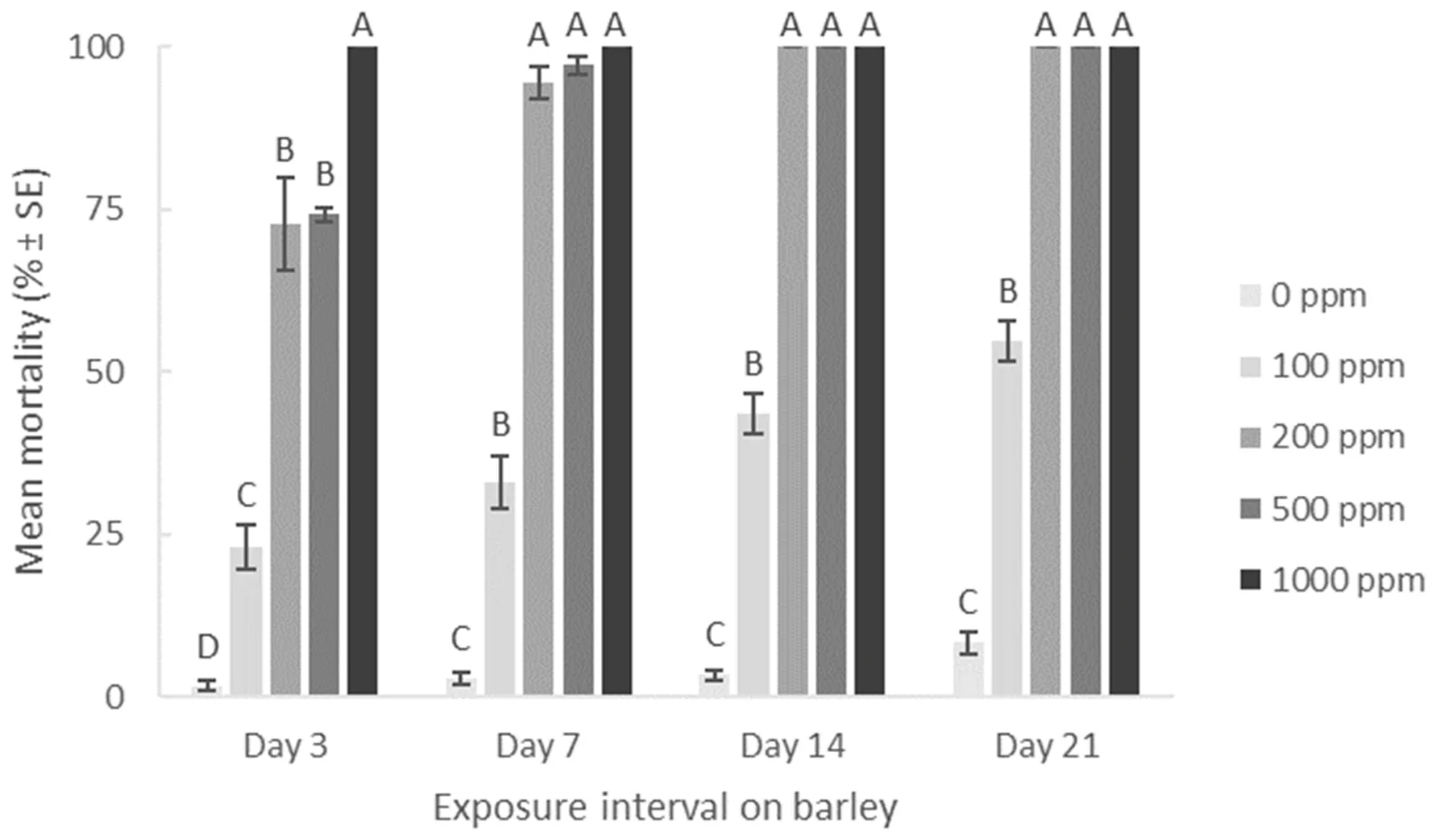
Mean mortality (% ± SE) of *T. confusum* after 3, 7, 14 and 21 d of exposure to barley treated with 0, 100, 200, 500 and 1000 ppm of ChinCheX^®^. Different letters among concentrations within each exposure interval indicate significant differences according to Tukey’s HSD test at α = 0.05.

Mortality of *T. confusum* on wheat and barley at the different concentrations and exposure intervals is presented in Figure 3 and Figure 4, respectively. Complete mortality of *T. confusum* on wheat treated with 500 and 1000 ppm was observed within the first 3 d of exposure, whereas 200 ppm achieved 100% mortality after 7 d (Figure 3). In contrast, mortality at 100 ppm remained significantly lower and did not exceed 35% by the end of the experimental period (21 d) (Figure 3). On barley, only the 1000 ppm treatment caused complete mortality of *T. confusum* within the first 3 d, whereas both 200 and 500 ppm achieved the same rates after 7 d of exposure (Figure 4). The lowest concentration, 100 ppm, achieved only 55% mortality after 21 d of exposure. In both commodities, survival of *T. confusum* in the control treatments remained high, with mortality ranging from 3.3 to 8.3% by the end of the exposure period.

Poisson GLM showed that progeny production of both *O. surinamensis* and *T. confusum* was significantly affected by concentration, whereas commodity and the commodity × concentration interaction were not significant (Table 2). Thus, progeny production in both species was determined primarily by insecticidal concentration rather than commodity type.

**Figure 5:**
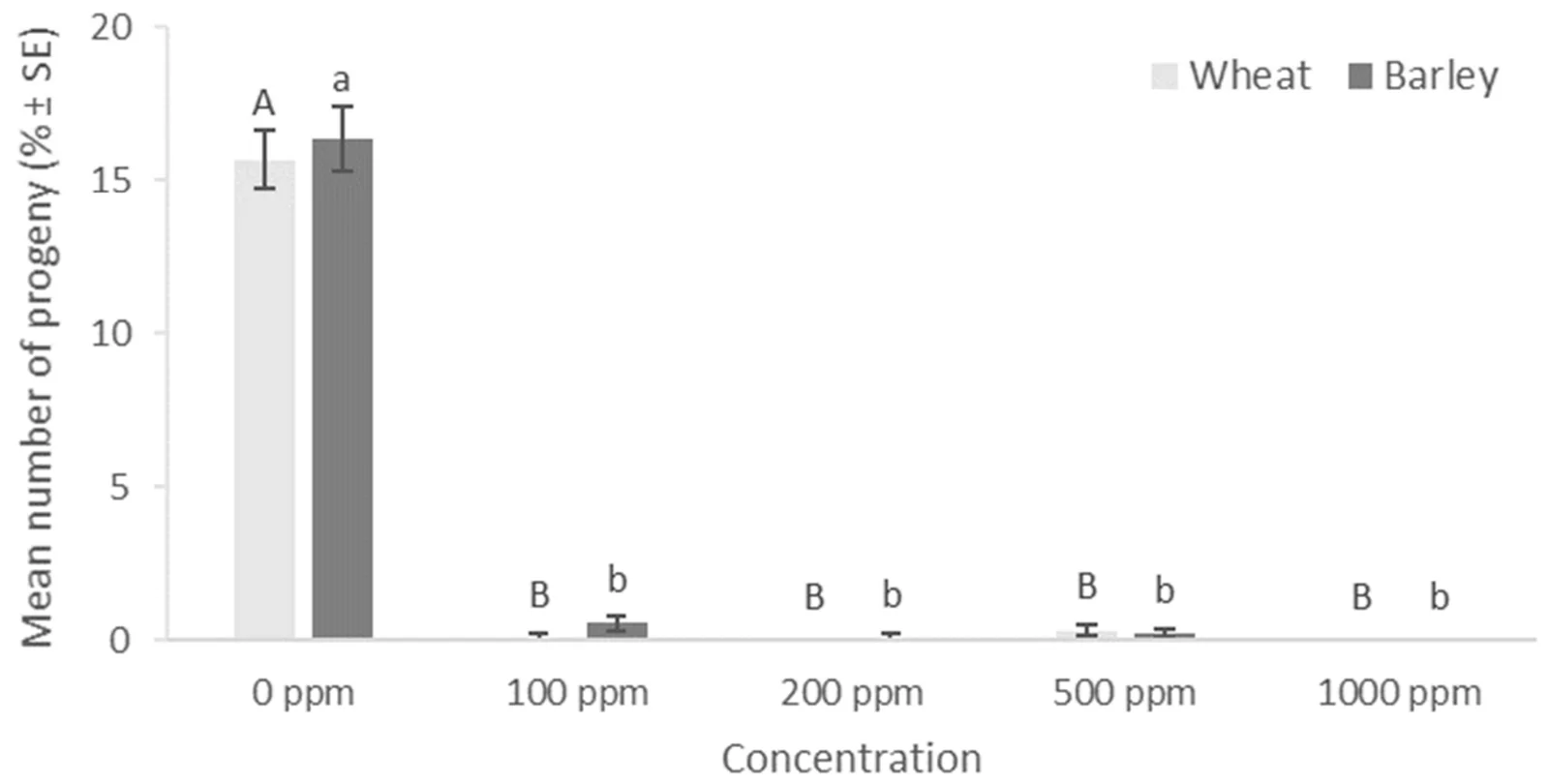
Mean number of progeny (±SE) per vial of *O. surinamensis* after 65 d of exposure to wheat or barley treated with 0, 100, 200, 500 and 1000 ppm of ChinCheX^®^. Different uppercase letters among the concentrations within wheat and lowercase letters within barley indicate significant differences according to Tukey’s HSD test at α = 0.05.

**Figure 6:**
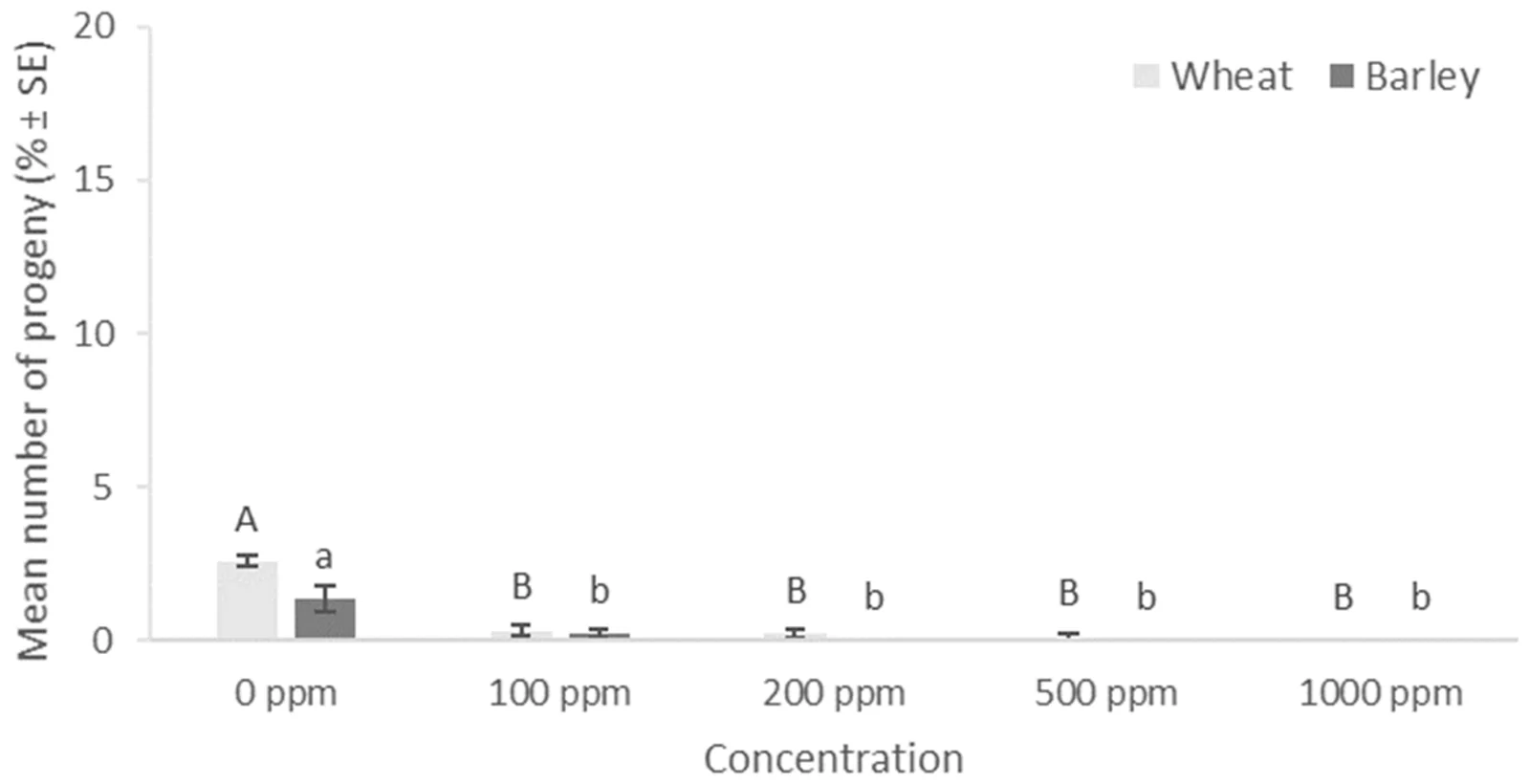
Mean number of progeny (±SE) per vial of *T. confusum* after 65 d of exposure to wheat or barley treated with 0, 100, 200, 500 and 1000 ppm of ChinCheX^®^. Different uppercase letters among the concentrations within wheat and lowercase letters within barley indicate significant differences according to Tukey’s HSD test at α = 0.05.

Mean progeny production of *O. surinamensis* and *T. confusum* on wheat and barley is presented in Figure 5 and Figure 6, respectively. Nevertheless, mean progeny production per vial was generally low, remaining below 1 individual in most insecticide-treated groups regardless of species or concentration (Figure 5 and Figure 6). In the control treatments, mean progeny production of *O. surinamensis* reached 16 individuals on both commodities (Figure 5). For *T. confusum*, although progeny production in the control was significantly higher than in the corresponding insecticide-treated groups, it also remained relatively low, averaging 3 individuals on wheat and 1 individual on barley (Figure 6).

### 3.2. Surface Treatment

Repeated-measures MANOVA indicated that mortality of both *O. surinamensis* and *T. confusum* was significantly influenced by surface, concentrations, exposure interval and their interactions (Table 3). These interaction effects were particularly pronounced in *T. confusum*, which showed much higher F values than *O. surinamensis*.

**Figure 7:**
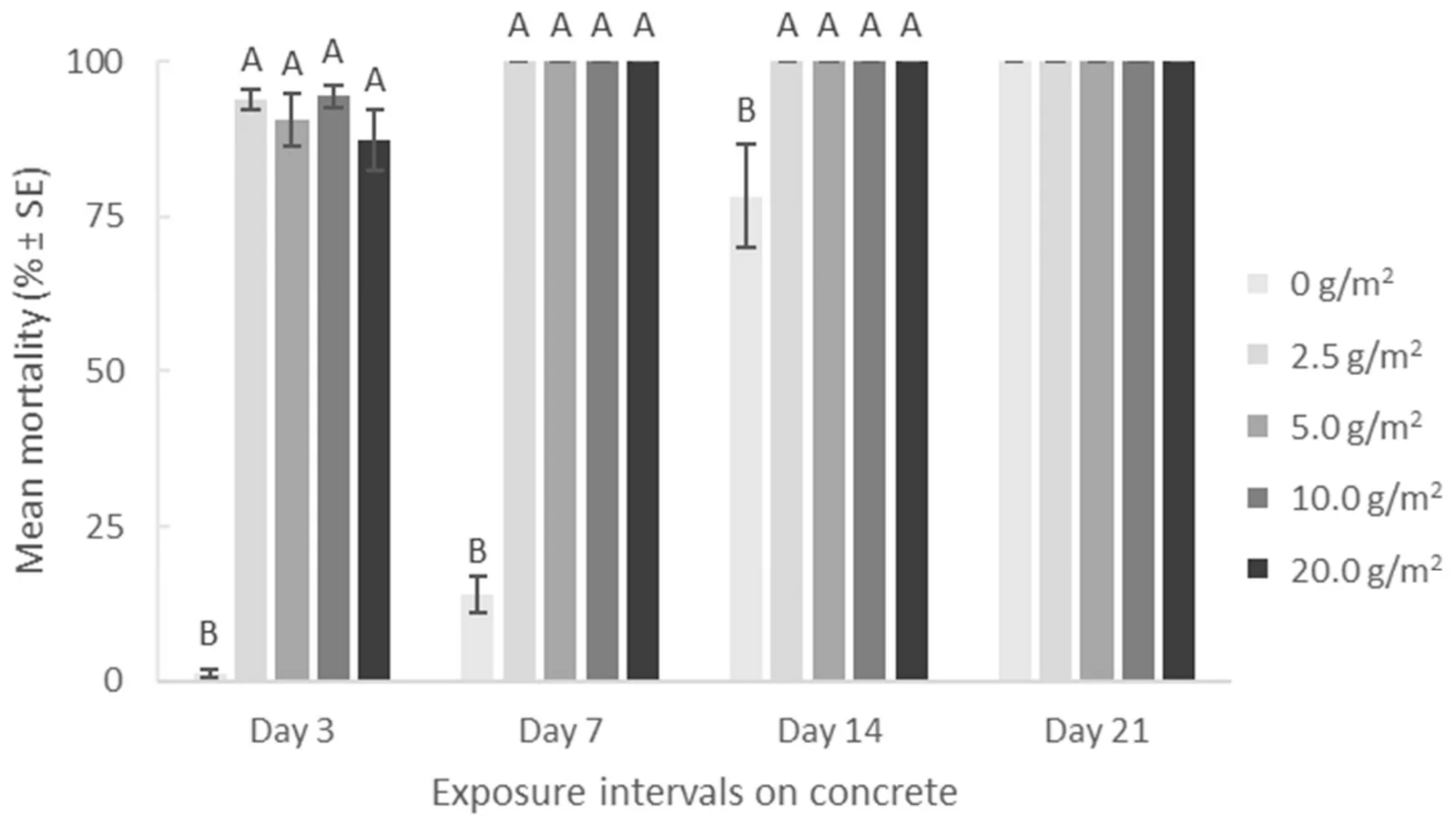
Mean mortality (% ± SE) of *O. surinamensis* after 3, 7, 14 and 21 d of exposure to concrete surface treated with 2.5, 5.0, 10.0 and 20.0 g/m^2^ of ChinCheX^®^. Different letters among concentrations within each exposure interval indicate significant differences according to Tukey’s HSD test at α = 0.05. Where no letters exist, no significant differences were found.

**Figure 8:**
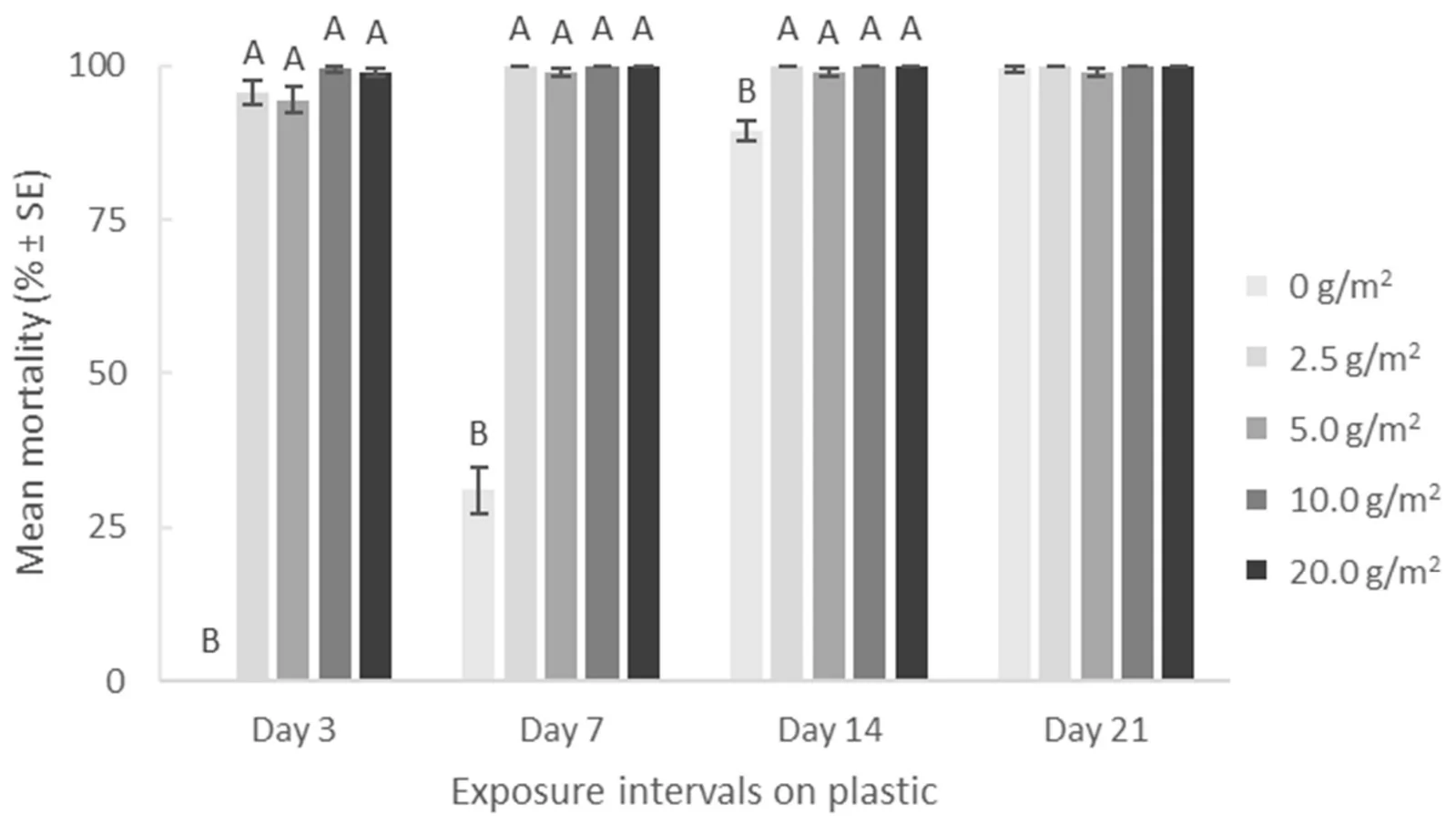
Mean mortality (% ± SE) of *O. surinamensis* after 3, 7, 14 and 21 d of exposure to plastic surface treated with 2.5, 5.0, 10.0 and 20.0 g/m^2^ of ChinCheX^®^. Different letters among concentrations within each exposure interval indicate significant differences according to Tukey’s HSD test at α = 0.05. Where no letters exist, no significant differences were found.

Mortality of *O. surinamensis* on concrete and plastic surfaces at the different application rates and exposure intervals is presented in Figure 7 and Figure 8, respectively. Mortality of *O. surinamensis* on concrete was high at all SiO_2_ concentrations, exceeding 85% after 3 d of exposure and reaching 100% at all treated concentrations by 7 d (Figure 7). In the untreated control, mortality remained low at 3 and 7 d, but increased substantially by 14 d and reached complete mortality by 21 d. A similar pattern was observed on plastic surfaces, where all silicon dioxide concentrations caused >94% mortality of *O. surinamensis* within the first 3 d of exposure (Figure 8).

**Figure 9:**
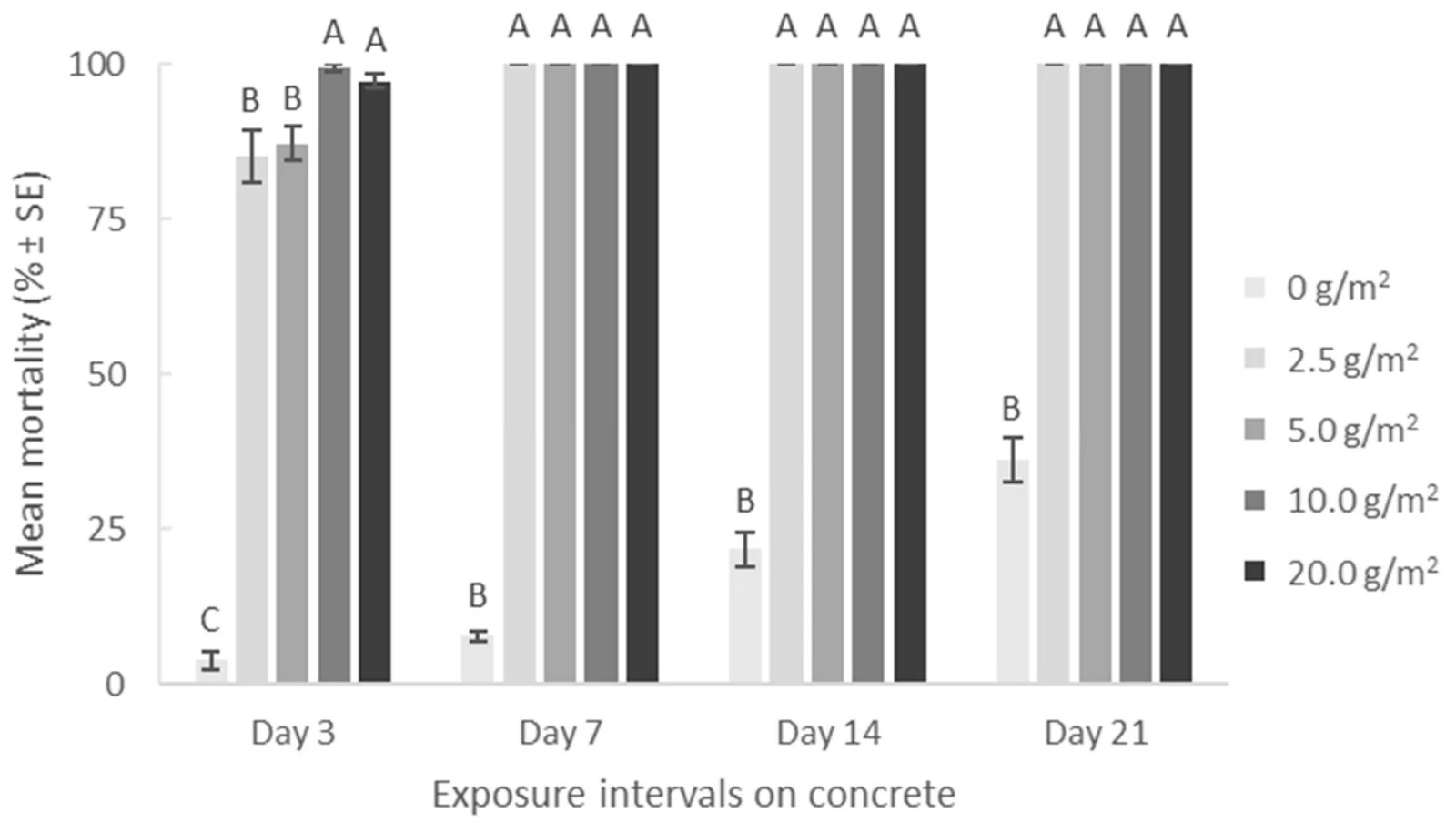
Mean mortality (% ± SE) of *T. confusum* after 3, 7, 14 and 21 d of exposure to concrete surface treated with 2.5, 5.0, 10.0 and 20.0 g/m^2^ of ChinCheX^®^. Different letters among concentrations within each exposure interval indicate significant differences according to Tukey’s HSD test at α = 0.05.

**Figure 10:**
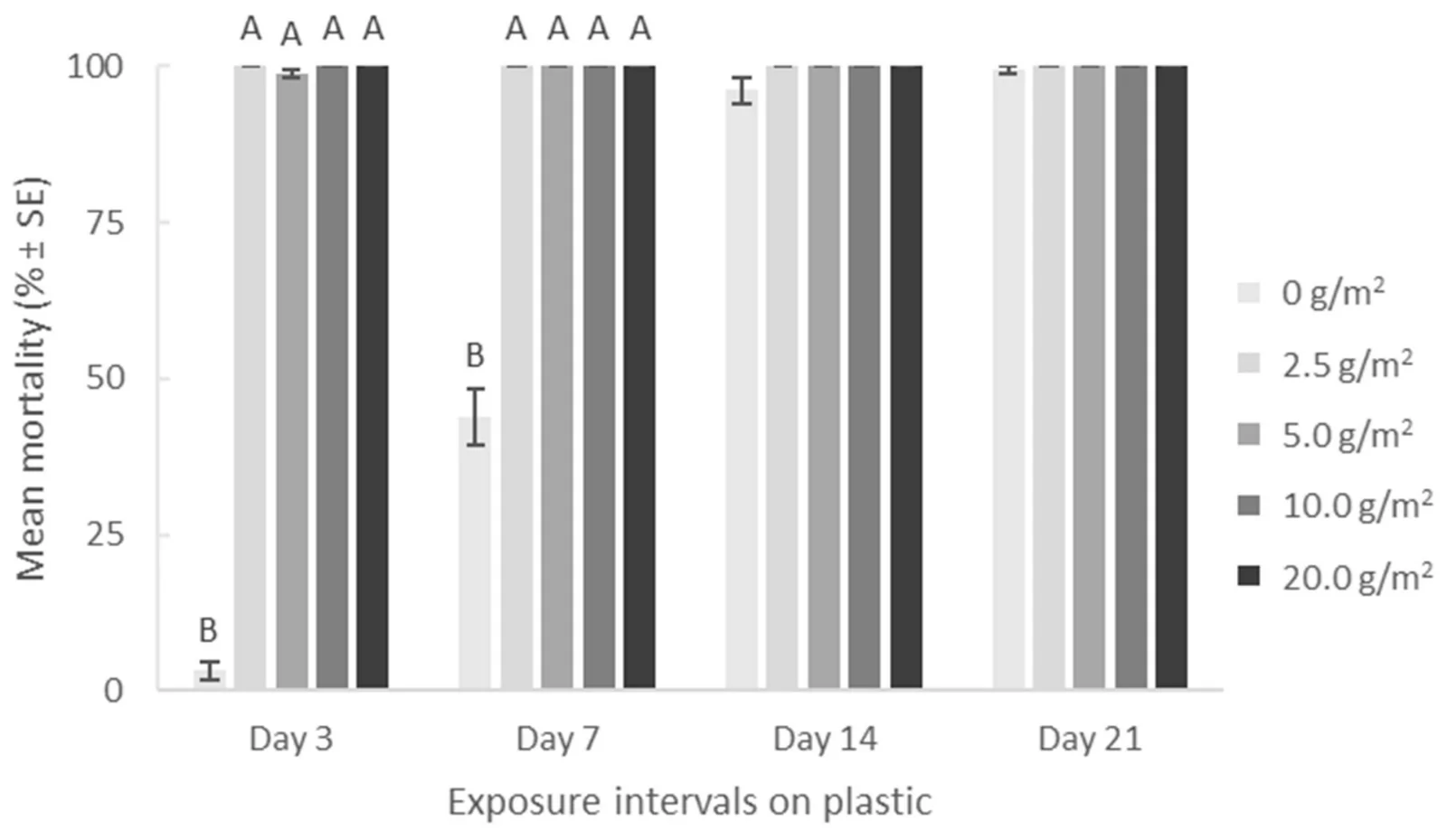
Mean mortality (% ± SE) of *T. confusum* after 3, 7, 14 and 21 d of exposure to plastic surface treated with 2.5, 5.0, 10.0 and 20.0 g/m^2^ of ChinCheX^®^. Different letters among concentrations within each exposure interval indicate significant differences according to Tukey’s HSD test at α = 0.05. Where no letters exist, no significant differences were found.

The corresponding mortality data for *T. confusum* on concrete and plastic surfaces are presented in Figure 9 and Figure 10, respectively. Mortality of *T. confusum* on concrete was likewise high at all concentrations, exceeding 85% after 3 d of exposure and reaching 100% at all treated concentrations by 7 d (Figure 9). Differences were observed only between the two lower concentrations and the two higher concentrations at Day 3 (Figure 9). On plastic surfaces, however, mortality exceeded 99% after 3 d regardless of the concentration (Figure 10).

In contrast to *O. surinamensis*, mortality of *T. confusum* in the untreated control remained significantly lower than that in the treated groups even after 21 d, but only on concrete surfaces. On plastic surfaces, by comparison, mortality in the untreated control increased markedly by 14 d and reached 100% by the end of the experimental period.

## 4. Discussion

The present study demonstrated that ChinCheX^®^, an amorphous silicon dioxide formulation, has strong insecticidal activity against adults of both *O. surinamensis* and *T. confusum* when applied either directly to grain or as surface treatment. Mortality generally increased with increasing concentration and exposure interval, while progeny production was low in all treated groups. These findings are in agreement with those of Quellhorst et al. [21], who reported that 200 and 500 ppm of the same formulation were effective for *R. dominica* and *S. granarius*, respectively. The efficacy of ChinCheX^®^ observed in the current study agrees with previous work on amorphous silica formulations. The present findings extend these observations to two additional economically important secondary pests, suggesting that ChinCheX^®^ has broad-spectrum potential as a grain protectant. Notably, complete mortality of *T. confusum* was achieved at concentrations of 200 ppm or higher, indicating that relatively low application rates may be promising for practical implementation, although validation under commercial storage conditions is still required.

In the grain-treatment bioassays, *O. surinamensis* was highly susceptible to ChinCheX^®^, as complete mortality was achieved even at the lowest concentration tested (100 ppm) after 21 d of exposure. In contrast, *T. confusum* exhibited lower susceptibility at this concentration, particularly on wheat, where mortality remained below 40% after 21 d. Differences in susceptibility among stored-product beetle species have been reported for diatomaceous earth and other silica-based formulations [23,24,25,26]. The greater susceptibility of *O. surinamensis* observed here may be associated with its smaller body size and greater movement through the treated grain mass, resulting in increased contact with dust particles. Arthur [27] indicated that *O. surinamensis* is highly susceptible to the Protect-It™ diatomaceous earth formulation and that mortality continues to increase even after individuals are removed from the treated commodity. Furthermore, Baliota and Athanassiou [28] reported considerable variation in the response of *O. surinamensis* to different Greek diatomaceous earth formulations, indicating that both insect species and formulation characteristics can strongly influence the efficacy of silica-based insecticides. For example, after 7 d of exposure at 1000 ppm, mortality of *O. surinamensis* ranged from only 1.1–2.7% with DE1, DE2 and DE3, whereas DE5 and DE6 caused 100% mortality. At 500 ppm, DE6 caused 96.1% mortality after 7 d and 100% mortality after 14 d. In the present study, ChinCheX^®^ caused complete mortality of *O. surinamensis* at 500 ppm after 7 d of exposure, showing a level of efficacy comparable to the formulations evaluated by Baliota and Athanassiou [28]. Commodity type significantly affected mortality levels in both species, although this effect was generally limited. Similar commodity-related differences have been documented in Saeed et al. [29] who tested the diatomaceous earth Grain-Guard in different stored product insects such as the psocid, *Liposcelis paeta* Pearman (Psocoptera: Liposcelidae), the rusty grain beetle, *Cryptolestes ferrugineus* (Coleoptera: Cucujidae), *R. dominica*, and the red flour beetle, *Tribolium castaneum* (Herbst) (Coleoptera: Tenebrionidae), on four grain commodities, wheat, rice, maize and sorghum and found that mortality was higher on wheat than on rice, maize and sorghum. Nevertheless, ChinCheX^®^ remained highly effective on both wheat and barley, indicating that its performance is relatively stable across different cereal commodities.

An important finding of the present study was the substantial reduction in progeny production. Although adult mortality is often the primary parameter used to evaluate grain protectants, progeny production is equally important from a practical perspective. In all treated groups, progeny production was almost completely suppressed, even at concentrations that did not always cause immediate adult mortality. For example, Quellhorst et al. [21] reported that ChinCheX^®^ significantly reduced progeny production of *S. granarius* and *R. dominica* on treated wheat, even at relatively low application rates. Similarly, Sakka et al. [30] found that diatomaceous earth Detech^®^ significantly reduced the progeny production of the invasive larger grain borer, *Prostephanus truncatus* (Horn) (Coleoptera: Bostrychidae), on maize, particularly at concentrations of 2000 and 3000 ppm. However, substantial numbers of offspring were still produced even at the highest concentration tested, indicating incomplete population suppression. More specifically, progeny production decreased from approximately 98 individuals per vial in the untreated control to 53.3 and 32.9 individuals per vial at 2000 and 3000 ppm, respectively. In contrast, the near-complete suppression of progeny production observed in the present study suggests that ChinCheX^®^ may provide a greater level of long-term population control. In the case of *T. confusum*, progeny production was relatively low even in the untreated grains. This can be explained by the fact that *T. confusum* is a secondary colonizer of stored grains and generally develops better on damaged or processed cereal products than on intact kernels. Therefore, the intact wheat and barley used in the present bioassays may have limited its progeny production. In addition, the survival of some parental adults in the treated grains did not result in increased progeny production under the conditions of the present study. The surface-treatment bioassays revealed even greater efficacy than the grain treatments. Mortality exceeded 85% after only 3 d of exposure in most cases and reached complete mortality within 7 d regardless of surface type or concentration. Similar rapid responses have been reported for silica-based formulations applied to structural surfaces, where direct contact between insects and dust deposits is maximized [4,16]. Interestingly, differences among concentrations became negligible after the first exposure interval, suggesting that even the lowest application rate tested may provide satisfactory control under laboratory conditions.

Overall, the present findings demonstrate that ChinCheX^®^ is highly effective against *O. surinamensis* and *T. confusum* adults both as a grain protectant and as a surface treatment. The high mortality rates, rapid activity on treated surfaces and almost-complete suppression of progeny production highlight its potential as a valuable component of integrated pest management programs for stored products. Future studies should evaluate its performance under commercial storage conditions and against additional stored-product insect species to further define its practical applicability.

## 5. Conclusions

Τhe present study demonstrates that the commercial silicon dioxide formulation ChinCheX^®^ is highly effective against adults of *O. surinamensis* and *T. confusum* when applied either as a grain protectant or as a surface treatment. In grain treatment bioassays, mortality increased with concentration and exposure time, with *O. surinamensis* showing high susceptibility even at the lowest tested concentration, whereas *T. confusum* required higher concentrations for complete control. Importantly, progeny production was almost completely suppressed in treated wheat and barley, indicating that the formulation can limit both adult survival and population growth in stored grains.

Surface treatments produced high mortality on both concrete and plastic surfaces, suggesting that ChinCheX^®^ may also be useful for treating storage surfaces where beetles move or shelter. Overall, these findings support the potential inclusion of this amorphous silicon dioxide formulation in integrated pest management programs for stored products. Further studies under commercial storage conditions, with different commodities, surface types, and longer residual efficacy, are needed to confirm its practical applicability.

## Figures and Tables

**Table 1:** Repeated measures MANOVA parameters for main effects (commodity and concentration) and associated interactions for mortality levels of *O. surinamensis* and *T. confusum* on 0, 100, 200, 500 and 1000 ppm of ChinCheX^®^ (error df = 80 for each insect species).

Insect Species	*Oryzaephilus surinamensis*			*Tribolium confusum*		
Source	F	df	*p*	F	df	*p*
Between interactions	416.7	9	<0.0001	817.4	9	<0.0001
Intercept	19,044.7	1	<0.0001	18,203.2	1	<0.0001
Commodity	26.5	1	<0.0001	15.0	1	<0.001
Concentration	929.7	4	<0.0001	1812.0	4	<0.0001
Commodity × Concentration	1.2	4	0.30	23.3	4	<0.0001
Within interactions *	13.9	27	<0.0001	9.7	27	<0.0001
Time	495.6	3	<0.0001	83.7	3	<0.0001
Time × Commodity	6.6	3	<0.001	2.4	3	0.06
Time × Concentration *	27.6	12	<0.0001	17.8	12	<0.0001
Time × Commodity × Concentration *	5.1	12	<0.0001	3.7	12	<0.0001

* Wilks’ Lambda approximate F value.

**Table 2:** Poisson GLM for the main effects (commodity and concentration) and associated interactions on progeny production of *Oryzaephilus surinamensis* and *Tribolium confusum* on two commodities (barley and wheat) at 0, 100, 200, 500 and 1000 ppm of ChinCheX^®^ (Pearson χ^2^/df = 0.56 for *Oryzaephilus surinamensis* and Pearson χ^2^/df = 0.47 for *Tribolium confusum*).

Insect Species	*Oryzaephilus surinamensis*			*Tribolium confusum*		
Source	df	χ^2^	*p*	df	χ^2^	*p*
Commodity	1	0.001	0.97	1	0.003	0.95
Concentration	4	133.7	<0.0001	4	15.2	<0.01
Commodity × Concentration	4	2.2	0.68	4	0.06	0.99

**Table 3:** Repeated measures MANOVA parameters for main effects (surface and concentration) and associated interactions for mortality levels of *O. surinamensis* and *T. confusum* after 3, 7, 14 and 21 d of exposure to concrete or plastic surface treated with 2.5, 5.0, 10.0 and 20.0 g/m^2^ of ChinCheX^®^ (error df = 80 for each insect species).

Insect Species	*Oryzaephilus surinamensis*			*Tribolium confusum*		
Source	F	df	*p*	F	df	*p*
Between interactions	342.6	9	<0.0001	1407.7	9	<0.001
Intercept	69,263.1	1	<0.0001	142,942.4	1	<0.001
Surface	11.3	1	<0.01	488.0	1	<0.001
Concentration	764.8	4	<0.001	2718.7	4	<0.001
Surface × Concentration	3.1	4	0.01	326.7	4	<0.001
Within interactions *	37.4	27	<0.001	30.3	27	<0.001
Time	725.6	3	<0.001	196.3	3	<0.001
Time × Surface	7.8	3	<0.001	19.7	3	<0.001
Time × Concentration *	78.7	12	<0.001	39.4	12	<0.001
Time × Surface × Concentration *	4.4	12	<0.001	22.2	12	<0.001

* Wilks’ Lambda approximate F value.

## Data Availability

The original contributions presented in this study are included in the article. Further inquiries can be directed to the corresponding author.
